# A Perifacial EMG Acquisition System for Facial-Muscle-Movement Recognition

**DOI:** 10.3390/s23218758

**Published:** 2023-10-27

**Authors:** Jianhang Zhang, Shucheng Huang, Jingting Li, Yan Wang, Zizhao Dong, Su-Jing Wang

**Affiliations:** 1 School of Computer Science, Jiangsu University of Science and Technology, Zhenjiang 212003, China; 211110701118@stu.just.edu.cn (J.Z.); schuang@just.edu.cn (S.H.); 2 CAS Key Laboratory of Behavioral Science, Institute of Psychology & Department of Psychology, University of the Chinese Academy of Sciences, Beijing 100101, China; lijt@psych.ac.cn (J.L.); wangyan1@psych.ac.cn (Y.W.); dongzz@psych.ac.cn (Z.D.); 3 Department of Psychology, University of the Chinese Academy of Sciences, Beijing 101408, China

**Keywords:** facial-muscle movement, electromyography, support vector machine, random forest, backpropagation neural network

## Abstract

This paper proposes a portable wireless transmission system for the multi-channel acquisition of surface electromyography (EMG) signals. Because EMG signals have great application value in psychotherapy and human–computer interaction, this system is designed to acquire reliable, real-time facial-muscle-movement signals. Electrodes placed on the surface of a facial-muscle source can inhibit facial-muscle movement due to weight, size, etc., and we propose to solve this problem by placing the electrodes at the periphery of the face to acquire the signals. The multi-channel approach allows this system to detect muscle activity in 16 regions simultaneously. Wireless transmission (Wi-Fi) technology is employed to increase the flexibility of portable applications. The sampling rate is 1 KHz and the resolution is 24 bit. To verify the reliability and practicality of this system, we carried out a comparison with a commercial device and achieved a correlation coefficient of more than 70% on the comparison metrics. Next, to test the system’s utility, we placed 16 electrodes around the face for the recognition of five facial movements. Three classifiers, random forest, support vector machine (SVM) and backpropagation neural network (BPNN), were used for the recognition of the five facial movements, in which random forest proved to be practical by achieving a classification accuracy of 91.79%. It is also demonstrated that electrodes placed around the face can still achieve good recognition of facial movements, making the landing of wearable EMG signal-acquisition devices more feasible.

## 1. Introduction

Facial muscle movements are a means of nonverbal communication that can convey emotions, intentions and messages through the movements of the eyes, eyebrows, lips and other facial muscles. They have essential applications in emotion recognition [1,2], human–computer interaction [3,4,5], psychotherapy [6], acting [7] and socialization [8]. For example, in acting, actors can use facial movements to express the emotions and characteristics of their roles; in psychotherapy, facial action can be used as a cue to help psychologists understand patients’ inner feelings and emotions.

There are two main traditional facial-movement-recognition methods, facial-image analysis [9,10] and video analysis [11,12], which rely on computer-vision algorithms. So far, computer-vision methods have achieved a high recognition performance for this task. However, there are high requirements for the recording environment. The recognition accuracy will be affected by the camera angle, image resolution and lighting conditions. To avoid the impact of these external factors, physiological signals, such as electromyography (EMG), electroencephalography (EEG), electrocardiography (ECG), etc., become another means of facial movement detection.

EMG refers to the electrical activity generated when the muscle motor unit in the muscle contracts and expands under the control of the nervous system [13]. These electrical activities objectively describe the anatomical and physiological characteristics of the muscle. According to different collection methods, electrodes can be divided into invasive and non-invasive. The invasive acquisition method needs to insert needle-shaped electrodes into the muscle. Although they can avoid signal crosstalk between muscles and obtain more accurate signal changes, needle-shaped electrodes penetrate the skin and could easily cause trauma to the skin. Conversely, the non-invasive surface electromyography (sEMG) signal-acquisition method involves placing a metal electrode on the skin surface of the measured muscle source. It measures the weak voltage generated during muscle contraction. This non-invasive approach can capture temporal or intensity information related to superficial muscle activation. This collection method is safer and easier to operate, so most experiments use the non-invasive sEMG collection method. For example, when measuring the EMG signal generated via facial movements for smiling, electrodes are placed on the skin surface of the zygomaticus major muscle; for the measurement of frowning, the electrodes are placed on the skin surface of the corrugator supercilii muscle.

In recent years, many efforts have been devoted to developing EMG signal-acquisition systems, of which some commercial devices [14,15] are representative. Still, they are generally expensive and have a limited number of channels. In addition, there is still room for further improvement in resolution, portability and data-transmission methods. To solve these problems, scholars have been actively exploring new solutions. For example, Fuentes et al. [16] designed a low-cost EMG sensor and compared it with a commercial Delsys system. In the study, the authors used three indicators to estimate the similarity between the output signals of two systems. The results show that the designed low-cost and commercial systems agree well. Yang et al. [17] designed a low-cost, high-sampling-rate and multi-channel EMG acquisition system, which uses wearable EMG sensors combined with a microcontroller unit and Wi-Fi module and the collected signal data are immediately forwarded to the host for further processing. In addition, it has been verified with commercial systems and the correlation coefficients are above 0.8. Zhu et al. [18] developed an eight-channel wireless acquisition system with a sampling rate of 2 KHz and a resolution of 24 bits and designed a hardware filter and a software filter to improve the accuracy of the measurement system. Moreover, the acquisition unit is packaged in a small 3D-printed shell, which is more convenient to wear. However, this device can only measure a few muscle movements simultaneously due to the limited number of channels. In summary, while previous studies have addressed some issues, challenges remain in efficiently assessing facial-muscle movements. Therefore, while ensuring low cost, we aim to enhance the performance of EMG acquisition devices further to realize capabilities such as multi-channel, high-resolution and wireless transmission.

EMG acquisition devices can be applied to facial-muscle-movement classification and have achieved high recognition rates [19,20,21,22,23]. But previously they placed the electrodes directly on the source of the facial muscles during the experiment. Although this placement ensures high-quality acquisition of EMG signals, it also brings some troubles: electrodes, instruments and tapes will give the subject an unnatural feeling and the electrodes’ weight will inhibit facial movements. In addition, the electrodes attached to the face may be displaced by muscle movement. To solve the problems mentioned above, A. Gruebler et al. [24] proposed a wearable device that utilizes EMG signals for facial-expression detection. In their study, the authors investigated the placement of electrodes on the face to determine the optimal method for signal acquisition. The experimental results demonstrated that the placement of the distal electrode achieved an average recognition rate of 98% for smiles and 96% for frowns. Although they only recognized two facial expressions, the results showed that the recognition performance could also be high with respect to placing the distal electrodes. Another study by Monica et al. [25] employed distal EMG signals and computer-vision techniques to detect smile action units while ensuring the electrodes did not obstruct the face. The results displayed an accuracy of 81% for AU6 (cheek Lift) and 82% for AU12 (the corners of the mouth pull up). Regrettably, they use limited channels and fewer facial movements were recognized. In this paper, we increased the number of channels for acquiring EMG signals and the number of facial movements identified.

Based on the previous discussion, we designed an EMG signal-acquisition system for capturing the movement signals of facial muscles, including two main components: (1) a multi-channel EMG signal-acquisition device and (2) signal-receiver software with real-time display and data processing. To verify the reliability and practicality of the system, we conducted two experiments: a comparison with a commercial system for reliability assessment, and categorizing facial-muscle movements to demonstrate the practicality. The main contributions of this paper are as follows:

(1) The proposed EMG signal-acquisition system is affordable, with numerous channels, enabling measurement across various facial regions. Its wireless transmission enhances portability and leaves an expansion interface so other users can use it to realize more data-acquisition channels. In addition, the device is equipped with hardware unipolar and bipolar switching.
(2) We propose a multichannel EMG acquisition scheme around the face that simultaneously acquires multiple facial-muscle movements, providing the possibility of a realistic and natural facial muscle assessment.
(3) We collected 484 EMG samples and classified muscle actions using traditional machine learning and neural networks. The experiment showed machine learning’s superiority in classifying muscle action from EMG signals, with high recognition rates even for electrodes not directly placed on the facial-muscle source.

The rest of this paper is organized as follows. Section 2 describes the hardware and software design of the EMG acquisition system; Section 3 presents the details of the experiments comparing the EMG acquisition system proposed in this paper with a commercial system; Section 4 describes the experiments on facial-movement recognition; Section 5 discusses the features and drawbacks of the EMG signal-acquisition system; Section 6 summarizes the paper and discusses future work.

## 2. Design of the Proposed System

### 2.1. Hardware System

**Figure 1:**
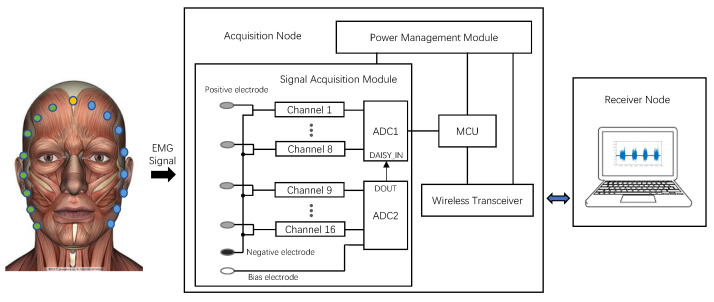
Schematic diagram of the measurement system architecture.

In this paper, we propose a multi-channel flexible configuration EMG signal-acquisition system and the overall framework diagram of the system is show in Figure 1. The system can be divided into two main nodes: acquisition node and receiver node. The acquisition node is composed of four main hardware components: a signal-acquisition module, a microcontroller unit (MCU), a power-management module and a wireless transmitter. The signal-acquisition module is designed to acquire EMG signals from the surface of the human body, amplify them and convert them into digital signals. The STM32F429, as a microprocessor unit (MCU), controls both the signal-acquisition module and the wireless transmitter. The ESP32C3-MINI1 wireless transmitter is responsible for wirelessly transmitting processed data via the MCU to the receiver node. The power-management module powers the entire system. The receiving node is mainly responsible for receiving, processing and displaying the data transmitted from the acquisition node.

#### 2.1.1. Signal-Acquisition Module

The signal-acquisition module comprises two analog-to-digital converter (ADC) chips, each of them has eight data-acquisition channels and one reference channel. Each channel includes positive and negative electrodes. A low-pass filter circuit is also included in each channel to eliminate high-frequency interference. To protect the chips from electrostatic breakdown, a diode is connected in parallel at the input end of each electrode.The ADS1299, produced by Texas Instruments, is used as the ADC due to its low noise, low power consumption, multi-channel, simultaneous-sampling, 24-bit delta-sigma (ΔΣ) ADC with an integrated programmable gain amplifier (PGA). The data samples per second (SPS) can be set between 250SPS and 16kSPS [26]. In addition, the daisy chain mode allows multiple chips to be connected in series to realize more data-acquisition channels and we have retained the expansion interface used in this module. Multiple EMG signal-acquisition modules can be connected in series using this interface. Due to the limitation of Wi-Fi transmission rate in this design, we can connect up to 4 EMG signal-acquisition modules in series into 64 data-acquisition channels at a sampling rate of 1 KHz. If there are better wireless transmission solutions in the future, we can realize more data-acquisition channels. The flexibility and extensibility of this design allows our system to meet the demand for a large number of data-acquisition channels in many different application scenarios.

Apart from the realization of multiple channels, the flexible use of unipolar and bipolar was also taken into account in our design. Each data-acquisition channel has positive electrode and negative electrode interfaces in the hardware design of the EMG acquisition module. When using the unipolar acquisition method, connect all the negative electrodes with jumper caps to complete the unipolar acquisition.

#### 2.1.2. Microcontroller Unit

As the core component of the signal-acquisition system, the controller has two main responsibilities. First, it sends instructions via the Serial Peripheral Interface (SPI) to control the registers of the ADS1299 chip, which allows adjustment of the operation mode, sampling rate and gain of each channel. Subsequently, it processes the data collected via the ADS1299, encapsulates it in a TCP/IP data frame format and then sends it to the wireless transmitter via SPI. Communication between the controller, ADS1299 and the wireless transmitter in our designed acquisition system is carried out through SPI. To accommodate the need for a more extensive data buffer space when collecting data with multiple ADC chips, we chose the STM32F429 core board as the controller. This board contains a higher-performance Cortex M4 core with a maximum operating frequency of 180 MHz, 256 KB of on-chip Static Random Access Memory (SRAM), 32 MB of external Synchronous Dynamic Random Access Memory (SDRAM), 6 SPIs and two Direct Memory Access (DMA) controllers (16 channels in total) [27]. Moreover, the small size of the core board, only 65 mm × 45 mm, makes it convenient for use in various projects while meeting our data cache space and fast data conversion requirements.

#### 2.1.3. Power-Management Module

A lithium battery is used to power all the modules, improving portability and enhancing the system’s anti-interference performance. The power supply voltages required by each module are as follows: 3.3 V for the controller and wireless transmitter and 3.3 V and 5 V for the signal-acquisition module. The lithium battery is connected to the circuit to generate a 3.7 V voltage, which is then passed through the HX4002 boost chip to generate a 5 V voltage. The 5 V voltage is passed through the LDO-TPS73201 chip to generate a 3.3 V power supply.

#### 2.1.4. Wireless Transmitter

Wireless communication technology is a widely used and necessary method for long-distance data transmission. In our system, the wireless transmitter serves two functions: (1) receiving and responding to commands from the controller and (2) forwarding the data processed by the controller to the PC for real-time waveform display. The amount of data generated by the acquisition device per second can be calculated using the following formula:

(1) D=N×fs×R

where *D* represents the data collected by the device per second in bits; *N* is the number of channels; fs denotes the sampling frequency and *R* is the data generated by one sampling per channel in bits. Since the ADS1299 uses a 24-bit ADC, in data processing, the 24 bits of each channel are changed to 32 bits to facilitate subsequent data conversion, so the value of *R* is 32. Therefore, when the system has 16 channels with a sampling rate of 1 KHz, the data volume collected by the device per second is 512 Kb/s. In practical applications, the actual transmission speed is affected by various factors in TCP transmission, such as frame structure, including headers, incremental bits, parity bits and packet retransmission overhead. In our case, the sampling rate of 16 channels is 1 KHz and the actual transmission speed should be around 620 Kb/s to avoid these interferences. To meet wireless transmission speed requirements, we reprogrammed the firmware of the ESP32C3-MINI1 [28] according to the instructions [29]; the purpose is to convert the Wi-Fi communication interface from serial interface to SPI to improve the data-transmission speed. Additionally, the ESP32C3-MINI1 has the advantages of small size and low power consumption, which makes it suitable for various applications.

In order to verify that the wireless transmitter we used was compliant, we conducted a test. Regarding our experiment requirement, the current transmission distance is deemed the effective range when the packet error rate is less than 0.02%. Under outdoor conditions, when the system operates in the standard continuous data-acquisition mode, the proposed system achieves a maximum transmission distance of 15 m, with an average packet error rate of just 0.015%. Consequently, this system is capable of realizing an efficient wireless interaction effect.

### 2.2. Software System

**Figure 2:**
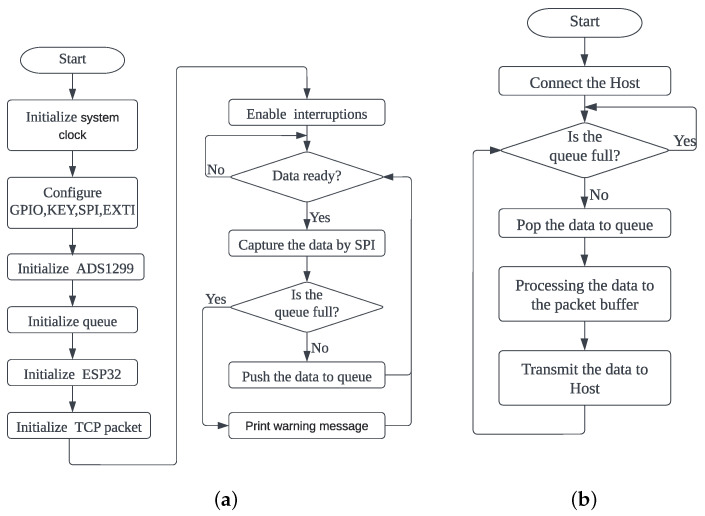
Program flow of the hardware program. (**a**) Program flow of the MCU; (**b**) Program flow of the Wi-Fi.

Our EMG signal-acquisition system’s software design comprises the hardware and graphical user-interface programs. As shown in Figure 2, the hardware program enables real-time data collection and wireless transmission. The graphic user-interface (GUI) program receives and stores data and real-time displays.

The hardware data-acquisition program that runs on the MCU performs two main functions: EMG signal acquisition and wireless data transmission. Figure 2a depicts the EMG signal-sampling program flow. Upon device startup, the main program initializes the system clock, GPIO ports, UART and SPI interfaces required by peripheral modules. Next, we initialize the registers of each ADS1299 and set the two ADC chips to daisy chain mode. In our experimental setup, a single channel has a sampling rate of 1 KHz, a gain of 24 and a resolution of 24 bits. Next, we set ESP32 to STA mode, connect to LAN and connect to the remote server. The EMG signal is converted into a digital signal by the ADC and placed in a circular queue instead of being sent directly to Wi-Fi. This method can reduce data loss caused by network instability. Later, the interruption is enabled for data collection. The ADS1299 chip sets the DRDY pin level low once the data are ready, generating a hardware interruption in the MCU. As illustrated in Figure 2b, when the interruption is triggered, the 16-channel EMG data are read continuously through SPI and placed into the queue buffer if the queue is not complete. When the connection between the wireless module and the remote server is established, if there are data in the queue, they are removed from the queue. After that, the data moved out are given a frame header, packet number, check digit, etc., encapsulated into a TCP data packet and eventually sent to the Wi-Fi module through SPI and forwarded to the remote server.

In the hardware part, we obtained the EMG data collected via the ADS1299 and sent them to the computer. At the same time, we wrote a simple GUI program using C++ under the QT platform, which mainly realizes three functions: Wi-Fi data reception and analysis, data storage and real-time display of data waveforms. QT Creator is a powerful cross-platform-integrated development environment compatible with Linux, Windows and macOS operating systems. The Qt, a C++ graphical user interface development framework, is used to develop a graphic user-interface program for the Windows operating system. Specifically, Qt Creator was chosen as the IDE and Qt 5.9 was selected as the framework to enable the efficient development of a user-friendly and responsive interface. After the program runs, the main thread creates a UI interface. Next, the user enters the port number to establish a TCP connection and waits for the Wi-Fi access request. After the TCP connection is established, a sub-thread is generated to receive, check and analyze the data packet and then store the processed data in the queue buffer. Finally, the main thread saves the data in a txt file and displays multi-channel waveforms.

## 3. Equipment-Comparison Experiment for Reliability

The primary purpose of this experiment is to verify the reliability of the EMG acquisition module proposed in this paper by comparing it with a commercial system MP160 produced by BIOPAC [30]. Table 1 lists the characteristics of the two devices. The commercial system consists of three modules: the EMG data-acquisition unit MP160, the advanced converter module HLT100C and a laptop computer installed with the supporting software AcqKnowledge 5.0. The advanced converter module and the laptop are connected via a network cable to form a local area network (LAN) and the data acquisition module MP160 sends the acquired data to the computer via Wi-Fi for reception, real-time display and storage.

### 3.1. Experimental Procedure

The purpose of this experiment is to verify the reliability of the device we designed. We recruited four subjects and to ensure that the signals collected by the two devices were comparable, we selected four symmetrical muscles on the face, namely the corrugator supercilii, orbicularis oculi, zygomaticus and orbicularis oris. We placed both systems on symmetrical muscle sources on the left and right sides of the participant’s face to ensure symmetrical electrode placement. Based on these four muscles, we selected four corresponding facial movements: frowning, blinking, smiling and pouting.

To ensure consistency between the acquisition methods of the two systems, we used the bipolar method to acquire EMG signals, mainly because the MP160 module collects signals in a bipolar manner. Moreover, the signal obtained in the bipolar way represents the potential difference between the positive and negative electrodes. This approach partially mitigates signal crosstalk among muscles and improves the received signal’s quality.

The experimental procedure is as follows:

(1) First, the subjects needed to understand the four facial movements for comparison and be able to display them.
(2) Second, we placed a pair of electrodes from both devices on top of the frowning muscles on the left and right sides of the participant’s face, respectively. Next, the subjects made three frowning movements were addressed. After that, we exchanged the position of the electrodes on the left and right sides of the participant’s face and asked the participant to repeat the frowning action three times. This approach can reduce the interference caused by the asymmetry of facial movements to a certain extent.
(3) Subsequently, the subjects completed the acquisition of EMG data for blinking, smiling and pouting in this way.

### 3.2. Index Evaluation

To better evaluate the difference between the commercial EMG acquisition equipment and the EMG acquisition equipment proposed in this paper, we selected four indicators of the Spearman correlation coefficient, energy ratio, linear correlation coefficient (LCC) and cross-correlation coefficient (CCC) to evaluate the correlation between the obtained data.

Spearman’s correlation is a non-parametric statistic used to assess the degree of association between two variables and is commonly used to measure a monotonic relationship between two variables. The Spearman correlation coefficient takes values between −1 and 1, where −1 indicates a completely inverse monotonic relationship, 0 indicates no monotonic relationship and 1 indicates an entirely positive monotonic relationship.

The energy ratio is usually used to compare the strength or energy of two EMG signals. The calculation formula is shown below. *E* denotes the energy and takes values ranging from 0 to 1, with 0 indicating no similarity and 1 denoting complete similarity and compares the magnitude of the energy level step by step. x(t) denotes the amplitude of the signals at the time point t.

(2) $$E=\int_{0}^{t}{\left|x(t)\right|}^{2}dt$$

Linear Correlation Coefficient (LCC): The LCC is calculated using the following equation: *x* represents the value of the signal captured by the system proposed in this paper and *y* represents the value captured by the commercial system. The values are taken in the range between −1 and 1. When LCC>0, it is a positive correlation. If LCC<0, it is a negative correlation. If LCC=0, there is no correlation between the two variables and it is an uncorrelated relationship.

(3) $$LCC=\frac{\sum \left(x-\bar{x}\right)\left(y-\bar{y}\right)}{\sqrt{\sum {\left(x-\bar{x}\right)}^{2}\sum {\left(y-\bar{y}\right)}^{2}}}$$

The cross-correlation coefficient (CCC) is a broad method of comparing EMG signals. CCC values range from −1 to 1, where −1 indicates a negative correlation and 1 is a positive correlation.

(4) $$CCC=\frac{n(\sum xy)-(\sum x)(\sum y)}{\sqrt{\left[n\left(\sum x^{2}\right)-{\left(\sum x\right)}^{2}\right]\left[n\left(\sum y^{2}\right)-{\left(\sum y\right)}^{2}\right]}}$$

### 3.3. Result

**Figure 3:**
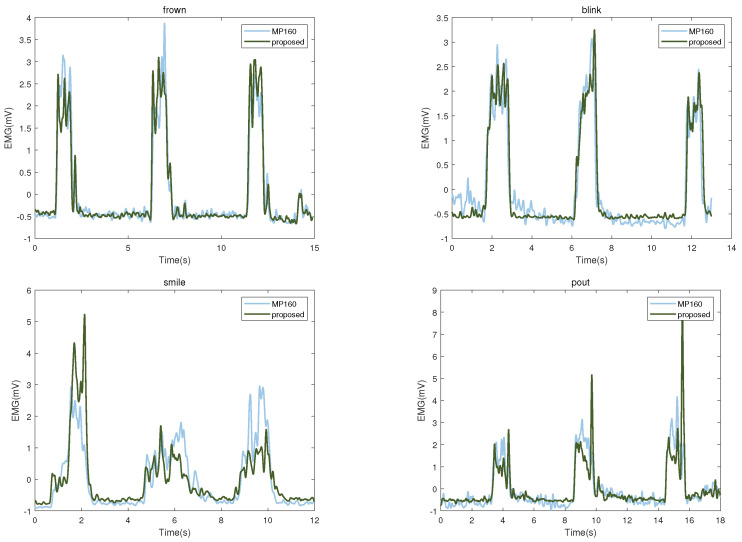
EMG signals from the commercial (blue) and the proposed device (green).

We used the correlation coefficient to evaluate the difference between our designed system and the commercial system after the same processing of the acquired EMG signal. Table 2 shows the evaluation results of the two systems under four facial-muscle movements. In the evaluation index of energy ratio, the similarity between the two is the highest. The similarity of the four indicators is above 70%, indicating that the signals collected by the two systems have good consistency. To show the difference between the two devices, we chose the comparison data when one of the subjects performed four facial-muscle movements. As shown in Figure 3, when frowning and blinking, there is less signal difference between the two devices; when smiling and pouting, our proposed device is more susceptible to sharp peaks.

## 4. EMG-Based Facial-Muscle-Movement Recognition

**Figure 4:**
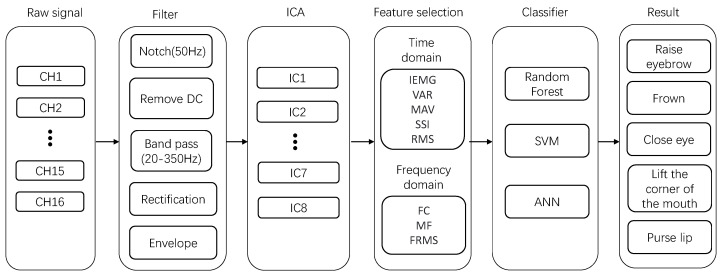
Structure of the EMG signal-processing scheme.

To demonstrate the practical value of the EMG acquisition system we designed, we conducted a facial-muscle-movement-recognition task. The task was different from previous facial-movement-recognition tasks using facial EMG (placing electrodes on top of the facial-movement source muscle to collect data). As shown in Figure 1, we placed electrodes around the face to collect crosstalk signals from the source muscle at the distal end using volume conduction between the muscles. The overall architecture of data analysis is shown in Figure 4. The specific experimental procedure is described below.

### 4.1. Electrode Placement

In our experiments, 18 electrodes (Ag/AgCl) were used. One is the reference electrode, another is the negative electrode and the rest were used to collect EMG signals. We placed all electrodes around the face to ensure unrestricted facial-muscle movement. The reference electrode is placed at a more neutral position relative to the face, i.e., behind the ear, while the negative electrode was placed at the forehead position. The remaining electrodes were placed on the left and right sides of the face, labeled L1–L8 and R1–R8. This electrode arrangement is referred to as a distal arrangement because the electrodes are not placed directly on the muscle sources of facial movements. Therefore, the acquired signals belong to the crosstalk signals propagated from the facial-muscle sources to the periphery of the face and such crosstalk signals may propagate in more than one direction. In addition, we considered the weight of the electrodes and the distance between the electrodes and finally chose to use 16 electrodes for signal acquisition.

### 4.2. Experimental Setup

We recruited ten healthy subjects, including eight males and two females, with an average age of 23.5 years, who actively participated in the experiment that lasted one hour. Before the start of the experiment, to reduce the impedance between the electrodes and the skin to obtain a clean sEMG signal, we cleaned the facial skin of the subjects using wipes containing alcohol. With the cleaning of the face completed, we followed the steps in Experiment 2.1 and distributed 16 electrodes evenly on both sides of the subject’s face, with eight electrodes on each side.

**Figure 5:**
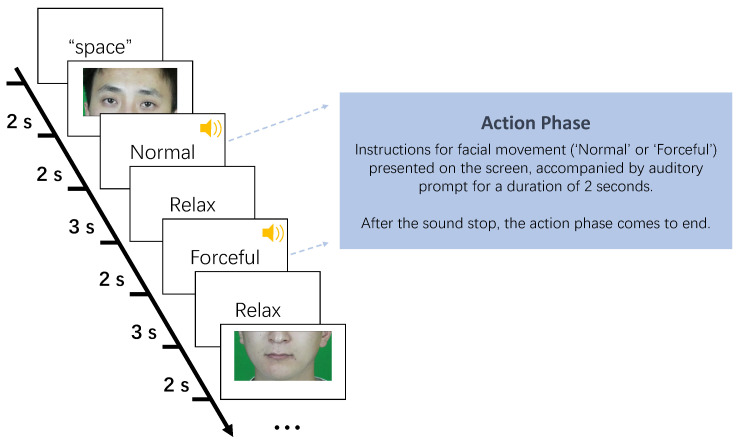
Data-Acquisition Experimental Procedure.

The experiment is described as shown in Figure 5. After the electrodes were attached, the subjects imitated the five facial-muscle movements (raising the eyebrows, frowning, closing the eyes, pursing the lips and lifting the corners of the mouth) shown on the computer screen. During the experiment, the computer screen displayed each facial-muscle action in two ways, normal and forceful. Then, we used the Psychopy program to instruct the subjects to display facial-muscle movements at these two intensities. The steps of the experiment are as follows:

Step 1: First, the program displays a picture of a facial-muscle movement on the computer screen for a duration of 2 s.

Step 2: Subsequently, an instructional word for facial-muscle-movement intensity (“normal”) was presented on the screen, accompanied by a sound.

Step 3: Subjects display the facial movement described in Steps 1 and 2 for 2 s. After that, the subjects had 3 s of muscle relaxation time.

Step 4: The screen then presented an instructional word (“forceful”) for the facial-muscle-movement intensity accompanied by a sound.

Step 5: Subjects followed Steps 1 and 4 to display the appropriate facial movement for 2 s. After the sound ended, they again returned to their natural state.

The above five steps are executed five times to form a block, capturing sEMG signals of different intensities for five facial-muscle actions. The whole experiment was divided into three blocks, with the same sequence of facial-muscle movements within each block and a 30 s rest period between different blocks. The experiment was divided into three blocks.

### 4.3. Data Pre-Processing

The pre-processing of the EMG data is realized via Matlab R2021b. The EMG data we collected first passed through a notch filter to remove power-line interference. After that, they passed through a fourth-order Butterworth bandpass filter with a frequency range of 20–350 Hz to preserve the main energy frequencies of the sEMG signals. Next, to make the EMG signals closer to the variations of the original muscle activity, we performed a direct current (DC) removal to improve the signal quality. Subsequently, we used a full-wave rectification to highlight the amplitude variation of the signal. Then, we used a low-pass filter to smooth the signal to obtain a linear signal envelope. Finally, the preprocessed data were used to obtain eight correlation components for feature extraction using the ICA algorithm.

**Figure 6:**
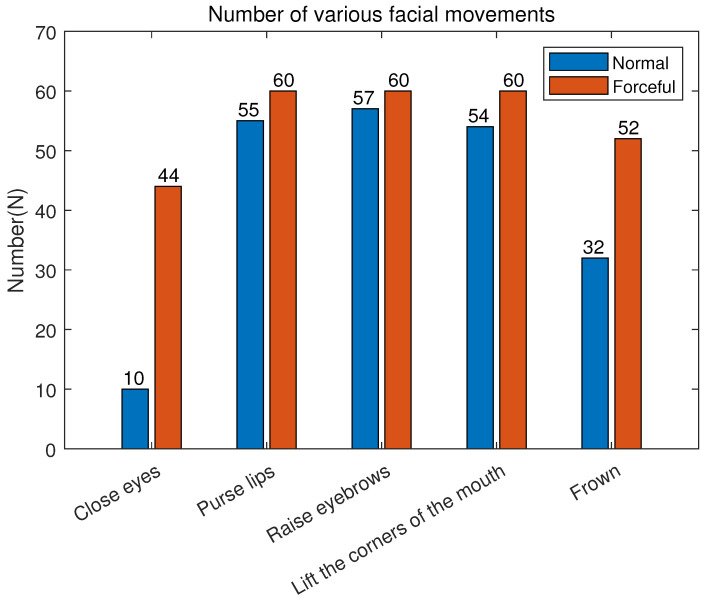
Number of various facial movements labeled.

During the experimental acquisition, we utilized a camera to record video of the subjects’ faces. This approach aimed to enhance labeling efficiency when associating EMG signals with specific facial-muscle movements. Figure 6 illustrates the number of labeled samples for five facial-muscle actions. The results indicate that, under forceful conditions, only three actions—puckering lips, raising eyebrows and lifting mouth corners—reached the desired 60 samples. Closing eyes had the fewest samples among the other facial actions, with only 44. Under normal conditions, the annotated sample count for all five facial actions did not reach the desired 60. The number of samples for frowning was about half of the ideal number. The number for closed eyes is the lowest, only 10.

Combined with the video analysis, we found that due to individual differences, some subjects had smaller movement amplitude when accomplishing closing eye and frowning movements. The electrodes placed around the face could hardly capture the signals. Although Figure 6 shows that we collected ten samples of closing eye (normal) data. By analyzing the video recordings of the subjects, we found that some of the subjects were actually closing their eyes (forceful) while performing the closing eye (normal). For data consistency, we still consider it a closing eye (normal). After excluding these invalid facial-muscle-movement data, we obtained 484 EMG signal samples for subsequent experimental analysis.

### 4.4. Source Separation

Due to the placement of electrodes around the face instead of directly on the muscle source responsible for facial movements, the measured facial muscle activity is obtained remotely. Consequently, when facial movements occur, the signal captured by an electrode can represent a combination of signals originating from multiple muscle sources to varying degrees. The Independent Component Analysis (ICA) algorithm can separate these mixed signals and identify their contributions. Applying the ICA algorithm makes it possible to disentangle the underlying muscle sources and enhance the accuracy of signal analysis in facial EMG studies.

Given a set of EMG signals (observations) ${\vec{x}}_{1}\left(t\right)$, ${\vec{x}}_{2}\left(t\right)$, ···, ${\vec{x}}_{n}\left(t\right)$, where t is time and n is the number of electrodes, they are assumed to be a linear mixture of independent components.

(5) $$\left(\begin{array}{c} {\vec{x}}_{1}\left(t\right)\\ \vdots \\ {\vec{x}}_{n}\left(t\right) \end{array}\right)=A\left(\begin{array}{c} {\vec{s}}_{1}\left(t\right)\\ \vdots \\ {\vec{s}}_{n}\left(t\right) \end{array}\right)$$

where A is the n×n mixing matrix, ${\vec{s}}_{1}\left(t\right)$, ${\vec{s}}_{2}\left(t\right)$, ···, ${\vec{s}}_{n}\left(t\right)$ is the original signal generated by the muscle source and the purpose of using ICA is to decompose the original signal ${\vec{x}}_{i}\left(t\right)$, i=1,2,3....n from the mixed signal ${\vec{s}}_{j}\left(t\right)$, j=1,2,3....n.

(6) $$\left(\begin{array}{c} {\vec{s}}_{1}\left(t\right)\\ \vdots \\ {\vec{s}}_{n}\left(t\right) \end{array}\right)=W\left(\begin{array}{c} {\vec{x}}_{1}\left(t\right)\\ \vdots \\ {\vec{x}}_{n}\left(t\right) \end{array}\right)$$

where $W=A^{-1}$ is an unmixing matrix of size n×n. This algorithm revealed the independent component (IC) weight corresponding to EMG in the mixed signal acquired by each electrode.

### 4.5. Feature Extraction

Feature selection is an essential step in EMG-based facial-muscle-movement recognition. To fully characterize the EMG signal, five time-domain features (IEMG, VAR, MAV, SSI, RMS) and three frequency-domain features (FC, MF, FRMS) are selected, as listed in Table 3. During the ICA separation process, the original 16-channel data are transformed, resulting in eight components. We systematically compute eight specific features for each component, resulting in 64 features for facial-muscle movement. These features are then combined and fed into a classifier for facial-muscle-movement classification.

### 4.6. Classification Implementation

Support Vector Machine (SVM) is a commonly used supervised learning method in machine learning, first proposed by Cortes and Vapnik in 1995 [31]. SVM is a powerful tool for dealing with small-sample, nonlinear problems. The most significant advantage of SVM is that it only needs a small number of samples to achieve good results. The core of SVM classification is to generate a hyperplane to classify the data. It involves introducing a kernel function to calculate the interval of the data pre-classification plane, aiming to seek a hyperplane that maximally separates different data classes. The mathematical expression of the hyperplane can be expressed as follows:

(7) wx−b=0.

where *x* is the vector on the hyperplane; w is the normal vector of the hyperplane; *b* is the intercept of the hyperplane. By solving the quadratic optimization problem, the values of *w* and b are obtained as:

(8) $$\begin{array}{c} \underset{\alpha }{min}\frac{1}{2}\sum_{i=1}^{N}\sum_{j=1}^{N}\alpha_{i}\alpha_{j}y_{i}y_{j}x_{i}^{T}x_{j}-\sum_{i=1}^{N}\alpha_{i}\\ s.t.\sum_{i=1}^{N}\alpha_{i}y_{i}=0,0\leq \alpha_{i}\leq C,i=1,2,\ldots N \end{array}$$

where *C* is the penalty parameter; *N* is the number of samples; α is the Lagrange multiplier vector; *y* is the label value. After we obtain the optimal solution $\alpha^{*}=\left(\alpha_{1}^{*},\alpha_{2}^{*},...,\alpha_{N}^{*}\right)$, we choose a component $\alpha_{j}^{*}$ of $\alpha^{*}$ that satisfies condition $0<\alpha_{j}^{*}<C$. We calculated $w^{*}$ and $b^{*}$.

(9) $$w^{*}=\sum_{i=1}^{N}\alpha_{i}^{*}y_{i}x_{i}$$

(10) $$b^{*}=y_{j}-\sum_{i=1}^{N}\alpha_{i}^{*}y_{i}x_{i}x_{j}$$

then the classification decision function formula is obtained as follows:

(11) $$f\left(x\right)=sign(\sum_{i=1}^{N}\alpha_{i}^{*}y_{i}\left(x\cdot x_{i}\right)+b^{*})$$

In this paper, a Gaussian kernel function is used as the kernel function of the SVM classifier, which extends the SVM from a linear problem to a nonlinear problem. In order to be able to perform multiple classifications, we adopt the “one pair redundancy” method. That is, we assume that the *m*th class of samples is a positive class and the other classes are regarded as negative classes and the final decision function is:

(12) $$f_{m}\left(x\right)=sign\left(\sum_{i=1}^{N}\alpha_{i}^{m}y_{i}K\left(x,x_{i}\right)+b_{m}\right)$$

Given a test sample x, take it into the above equation and take the maximum value as the classification result of SVM.

The random forest algorithm is an ensemble learning approach that combines bagging and random feature-selection techniques. Conceived by Breiman in 2001 [32], this method has entrenched itself as a staple within machine learning. It exhibits versatility, serving as a regression, prediction and feature-selection tool. Central to random forest’s mechanics is the assembly of a classifier through the orchestrated collaboration of multiple decision trees. These decision trees collectively undertake the training and prediction of samples. Each decision tree draws from an independent set of values, engendering diversity through employing a random vector. This vector, derived from a pre-defined probability distribution, shapes the distinctiveness of each tree. Following establishing a forest comprising a specified number of decision trees, the final prediction ensues through a straightforward majority-voting process. This algorithm’s sophistication lies in its ability to harness diverse decision trees, amplifying its predictive capabilities.

Backpropagation neural network (BPNN) is a common type of artificial neural network. It is a multi-layer feedforward neural network composed of an input, hidden and output layer. The training process of this network involves two key steps: forward propagation and backpropagation. During the forward-propagation phase, input data pass through the network layers sequentially, generating computed outputs. Subsequently, in the backpropagation phase, the network’s weights are adjusted using errors by comparing the actual outputs with the expected outputs. Through multiple iterations of these two steps, the network gradually refines its weights to approach the desired results. The BP neural network is renowned for its powerful learning and approximation capabilities and it finds extensive applications in areas such as pattern recognition, predictive analysis, classification tasks and more.

### 4.7. Result of the Facial-Muscle-Movement Recognition

In this experiment, the experimental design mentioned five facial-muscle movements at two different intensities, so we performed two categorization tasks. One was to classify the five facial-muscle movements (forceful) and the other was to classify ten facial-muscle movements (normal and forceful). The data used for classification had 484 samples, of which the samples of facial-muscle movements (forceful) had 276. Then, we chose SVM, random forest and BPNN as classifiers. In addition, to verify the reliability of the classification results, we used 80% of the data for training and 20% for testing randomly. Three metrics, accuracy, recall and F1 score, were used to evaluate the performance of the classifiers.

**Figure 7:**
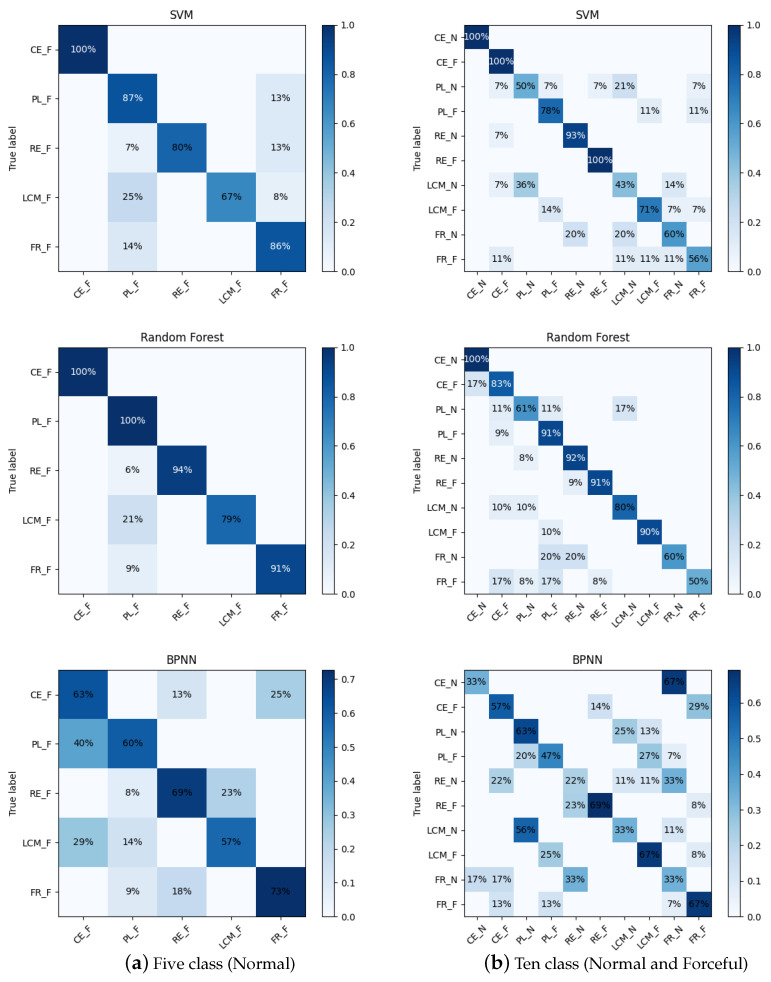
Confusion matrix for results of five facial-muscle movements and ten facial-muscle-movements recognition. CE_N: Close eyes (Normal); PL_N: purse lips (Normal); RE_N: Raise eyebrows (Normal); LCM_N: Lift the corners of the mouth (Normal); FR_N: Frown (Normal); CE_F: Close eyes (Forceful); PL_F: purse lips (Forceful); RE_F: Raise eyebrows (Forceful); LCM_F: Lift the corners of the mouth (Forceful); FR_F: Frown (Forceful).

Table 4 illustrates the classification results of all the models on the test set. Among the classification results of five facial-muscle movements, the accuracy rates of SVM, random forest and BPNN are 82.14%, 91.79% and 64.28%, respectively. Among the classification results of ten facial-muscle movements, SVM, random forest and BPNN have 71.13%, 77.31% and 52.57% correct classification rates, respectively. Figure 7 compares the confusion matrices of the three classifiers for the two classification tasks. The confusion matrix clearly lists the correct and incorrect classifications: the diagonal elements represent the number of correctly classified samples for each category and the elements not above the diagonal represent the number of incorrectly classified samples. Comprehensively comparing the two classification tasks, random forest has the best classification results compared to BPNN and SVM. We analyze the classification results of the random forest. Under the intensity of forcefulness, the highest correct classification rate is for closing the eyes and pursing the lips. The lifting of the corners of the mouth is sometimes incorrectly classified as pursing the lips because they have a similarity in the appearance of the facial movement. Raised eyebrows and frowns had some data incorrectly categorized as pursed lips, possibly due to problems with the data themselves. Among the results of the classification of ten facial-muscle movements (normal and forceful), normal eye closure achieved the highest correct classification rate. Partially forceful eye closure was classified as normal eye closure and partially normal pursing of the lips was classified as forceful pursing because subjects did not distinguish between normal and forceful when performing the movements. Partially normal pursing of the lips was also misclassified as normal lifting of the corners of the mouth. All other facial-muscle movements were misclassified in a small fraction of the data, with frowning having the worst results.

## 5. Discussion

In the past few years, EMG signals have played an essential role in the biomedical field. With the development of technology, many EMG signal-acquisition solutions have been designed. However, the commonly used EMG signal-acquisition devices in the market still have room for improvement in price, number of channels and expansion performance. For this reason, we have designed a low-cost, multi-channel, scalable device. Next, we will discuss this device more deeply based on its features and experiments.

In the design of this system, we employed the daisy chain mode of the ADS1299 chip to realize multi-channel data collection. In theory, this approach can support more channels, but it requires attention to several key considerations. Firstly, as the number of connected chips increases, the transmission delay between chips becomes more pronounced, so special data-transmission processing between chips is required to reduce the delay. Secondly, multi-channel data collection increases data volume, making the size of the controller’s memory particularly crucial. A larger memory provides a more extensive data buffer space, which aids in optimizing the performance coordination between data collection, processing and transmission, especially in dealing with the instability of network transmission. Lastly, it is important to note that the maximum Wi-Fi transmission speed in the current system is limited to 2.9 Mbps/s. We will explore better wireless transmission solutions to improve data-transfer efficiency in future designs.

In addition to expanding the number of channels of the EMG acquisition module, the module also considers the switching between unipolar and bipolar acquisition methods to increase the system’s flexibility. If the experiment only needs to measure the activity of a single muscle or a few muscles, bipolar acquisition is a better choice. Compared to unipolar, the bipolar method can effectively suppress signal crosstalk between different muscles. However, if the experiment needs to measure the entire facial expression, we recommend a unipolar configuration for the periphery of the face. The main reason for this is that the bipolar approach requires the use of more electrodes covering the facial-muscle sources, which may not only feel unnatural to the subject but may also interfere with the normal movement of the facial-muscle sources due to factors such as the weight and size of the electrodes.

In the facial-muscle-movement recognition experiment, we used a unipolar approach with 16 electrodes evenly distributed around the face. This electrode placement did not interfere with the movement of the facial-muscle sources and the subjects could show facial-muscle movements more naturally. However, this method also has some limitations because the electrodes are not directly placed on the facial-muscle sources, so the acquired signals may be relatively weak and even the signals of some facial-muscle movements are almost unable to be acquired, such as blinking (normal) and frowning (normal). In contrast, the EMG signals of the frontalis, orbicularis oculi and zygomaticus muscles, both in the normal state and under exertion, can be effectively captured according to the labeling results. These labeling results indicate that this electrode configuration is feasible for evaluating facial-muscle movements, further providing a solution for facial-expression analysis limited by small sample problems, such as composite expressions, pain studies and micro-expressions. Specifically, with the perifacial EMG device, we can efficiently annotate facial-muscle movements by temporal and spatial variations of facial-muscle signals without affecting facial-image acquisition. This annotation can provide a large amount of sample data to train intelligent analysis of facial expressions under specific tasks.

Moreover, some aspects of this collection method can be explored in depth, starting with the size of the electrodes. In the experiments, we used larger-size Ag/AgCl electrodes. If some smaller-sized flexible electrodes can be used in the future, more electrodes can be placed around the face, thus potentially acquiring finer signals of facial-muscle movements. Secondly, the distance of the electrodes from the facial-muscle source needs to be considered to ensure that the electrodes do not interfere with facial-muscle movement but also to ensure that the full range of facial-muscle-movement signals is captured. If used with computer-vision methods, special consideration needs to be given to the distance between the electrodes and the facial-muscle source. All these issues need to be gradually verified and thoroughly investigated in future experiments to further enhance the performance and application prospects of the EMG signal-acquisition system.

Eventually, we chose a dedicated laboratory for data acquisition, in which only the necessary equipment required for the experiment was kept to minimize the impact of electromagnetic interference, wireless signal interference, etc., on the results. However, there are some challenges in real-world applications, such as environmental diversity, complex wireless and electromagnetic interference in cities, hardware failure or performance degradation, etc., which may negatively affect the proper operation of the equipment. All of these issues will require continuous testing in the future to improve the circuit design of the device. It is also difficult to avoid being affected by the outliers, known as noise or disturbances. To minimize these effects, we can use a variety of methods. First, data-processing techniques, such as smoothing, filtering, interpolation, etc., can be utilized. Secondly, the effect of individual outliers on the results can be reduced by multiple measurements. Finally, we can optimize the algorithm of the signal-acquisition system to make it more robust and better resistant to the effects of noise and outliers.

## 6. Conclusions and Future Work

This paper presents the design and implementation of a high-resolution, 16-channel wireless EMG measurement system. The multi-channel approach allows the system to measure different muscle activities of an individual simultaneously, increasing the system’s flexibility and applicability. The acquired data can be transferred to a computer via Wi-Fi for display, storage and further analysis. In addition, we compared this designed acquisition system with a commercial device and the correlation between the signals acquired by both devices was above 70%, which illustrates the reliability of our designed device. Finally, we placed electrodes around the periphery of the face to capture crosstalk signals propagating to the edge of the face during facial-muscle movements. We compared three different classifiers to categorize five facial activities, with the random forest providing the best recognition. This study demonstrates the ability of this EMG signal-acquisition device by us to recognize facial movements using distal EMG.

In our future work, we will utilize the proposed EMG signal-acquisition device and its distal electrode configuration scheme to enhance the detection and recognition of facial-muscle movements, achieving a more comprehensive assessment of facial muscle activity. Our electrode-placement method does not obstruct the face, offering a novel approach to the design of wearable facial EMG signal-acquisition devices. Furthermore, due to its non-obstructive nature, this facial EMG signal-acquisition method can be multimodal integrated with computer vision, thereby enhancing the performance of facial-muscle-movement detection and recognition. This technology holds potential application value in hardware-based facial recognition computation and criminal investigation.

## Figures and Tables

**Table 1:** Characteristics of commercial and proposed systems.

Parameter	Proposed	Commercial
Number of channels (*N*)	16	2
Gain (*G*)	24	500
Sampling frequent ($f_{s}$)	1 K sps	1 K sps
A/D resolution	24 bits	16
Communication type	Wi-Fi	Wi-Fi
Power upply	3200 mAh Li battery	Cable
Signal-use time ($T_{sm}$)	5 h	
Dimensions	6.1 cm × 9.1 cm	
Weight	100.3 g	
Electrode material	Ag/AgCl	Ag/AgCl

**Table 2:** Comparison of correlation coefficients between our equipment and commercial equipment.

Exercise	Spearman	Energy Ratio	LCC	CCC
Frown	0.78	0.96	0.88	0.88
Blink	0.74	0.94	0.91	0.91
Smile	0.82	0.96	0.91	0.92
Pout	0.78	0.91	0.78	0.78

**Table 3:** EMG-based features from time domain (first five) and frequency domain (last three).

Name	Mathematical Formula	Description
IEMG	$IEMG=\sum_{i=1}^{N}\left|x_{i}\right|$	Signal power estimator: calculating the sum of absolute values of EMG signals.
VAR	$VAR=\frac{1}{N}\sum_{i=1}^{N}{\left(x_{i}-\bar{x}\right)}^{2}$	Distance between the number and the average.
MAV	$MAV=\frac{1}{N}\sum_{i=1}^{N}\left|x_{i}\right|$	Add up all absolute values and divide by length.
SSI	$SSI=\sum_{i=1}^{N}\left|x_{i}^{2}\right|$	The energy of electromyography.
RMS	$RMS=\sqrt{\frac{1}{N}\sum_{i=1}^{N}x_{i}^{2}}$	Amplitude-modulated Gaussian stochastic processes with RMS values related to constant and non-constant forces.
FC	$FC=\frac{\sum_{n=1}^{N}f_{n}S\left(n\right)}{\sum_{n=1}^{N}S\left(n\right)}$	Used to describe the signal in the spectrum of the larger component of the signal components of the frequency, reflecting the distribution of the signal power spectrum.
MF	$MF=\frac{\sum_{n=1}^{N}S\left(n\right)}{N}$	Frequency mean value.
FRMS	$FRMS=\sqrt{\frac{\sum_{n=1}^{N}f_{n}^{2}S\left(n\right)}{\sum_{n=1}^{N}S\left(n\right)}}$	Root mean square frequency.

**Table 4:** The results of categorizing facial movements on the test set.

Category	Classifier	Accuracy	Recall	F1-Score
five class	SVM	82.14%	83.80%	82.86%
	Random Forest	**91.79%**	**92.78%**	**91.31%**
	BPNN	64.28%	64.32%	63.96%
ten class	SVM	71.13%	75.09%	70.09%
	Random Forest	**77.31%**	**79.85%**	**76.07%**
	BPNN	52.57%	49.10%	48.82%

## Data Availability

Not applicable.
